# Deciphering the hormonal signalling network behind the systemic resistance induced by *Trichoderma harzianum* in tomato

**DOI:** 10.3389/fpls.2013.00206

**Published:** 2013-06-24

**Authors:** Ainhoa Martínez-Medina, Iván Fernández, María J. Sánchez-Guzmán, Sabine C. Jung, Jose A. Pascual, María J. Pozo

**Affiliations:** ^1^Department of Soil Microbiology and Symbiotic Systems, Estación Experimental del Zaidín, Consejo Superior de Investigaciones CientíficasGranada, Spain; ^2^Estación Experimental La Mayora, Consejo Superior de Investigaciones Científicas, MálagaSpain; ^3^Department of Soil and Water Conservation and Organic Waste Management, Centro de Edafología y Biología Aplicada del Segura, Consejo Superior de Investigaciones CientíficasMurcia, Spain

**Keywords:** *Botrytis* sp.,induced systemic resistance, jasmonic acid, phytohormone, priming, signaling, tomato, *Trichoderma* sp.

## Abstract

Root colonization by selected *Trichoderma* isolates can activate in the plant a systemic defense response that is effective against a broad-spectrum of plant pathogens. Diverse plant hormones play pivotal roles in the regulation of the defense signaling network that leads to the induction of systemic resistance triggered by beneficial organisms [induced systemic resistance (ISR)]. Among them, jasmonic acid (JA) and ethylene (ET) signaling pathways are generally essential for ISR. However, *Trichoderma* ISR (TISR) is believed to involve a wider variety of signaling routes, interconnected in a complex network of cross-communicating hormone pathways. Using tomato as a model, an integrative analysis of the main mechanisms involved in the systemic resistance induced by *Trichoderma harzianum* against the necrotrophic leaf pathogen *Botrytis cinerea* was performed. Root colonization by *T. harzianum* rendered the leaves more resistant to *B. cinerea* independently of major effects on plant nutrition. The analysis of disease development in shoots of tomato mutant lines impaired in the synthesis of the key defense-related hormones JA, ET, salicylic acid (SA), and abscisic acid (ABA), and the peptide prosystemin (PS) evidenced the requirement of intact JA, SA, and ABA signaling pathways for a functional TISR. Expression analysis of several hormone-related marker genes point to the role of priming for enhanced JA-dependent defense responses upon pathogen infection. Together, our results indicate that although TISR induced in tomato against necrotrophs is mainly based on boosted JA-dependent responses, the pathways regulated by the plant hormones SA- and ABA are also required for successful TISR development.

## INTRODUCTION

Root colonization by selected *Trichoderma* isolates has been reported to increase resistance to different types of pathogens in various plant species, both below and aboveground (reviewed in Harman et al., 2004). This biological control can be achieved by a direct effect of *Trichoderma* on plant pathogens (reviewed in Vinale et al., 2008); or indirectly through plant-mediated effects by improving the plant nutritional status (Shoresh and Harman, 2008) or through partial activation of the plant immune system (reviewed in Shoresh et al., 2010). Indeed, some competent *Trichoderma* strains can colonize plants roots without any damage to plant tissues but inducing changes in plant physiology and the plant defense system (Yedidia et al., 1999; Alfano et al., 2007; Chacón et al., 2007; Brotman et al., 2012; Mathys et al., 2012). As in other beneficial plant–microbe interactions, these changes could be associated with a regulatory strategy of the plant to limit microbial colonization of the “ beneficial invader” (Zamioudis and Pieterse, 2012).

Although a clear understanding of the *Trichoderma*–plant recognition process is lacking, several elicitors that can activate plant basal immunity have been described in *Trichoderma* including the ethylene (ET)-inducing xylanase (Hanson and Howell, 2004); the proteinaceous non-enzymatic elicitor Sm1 (Djonovic et al., 2006, 2007); or the 18mer peptaibols (Viterbo et al., 2007). Only a limited number of pattern recognition receptors able to recognize some of these *Trichoderma*-related microbe-associated molecular patterns (MAMPs) have been characterized so far (Ron and Avni, 2004; Petutschnig et al., 2010). During the “ asymptomatic” infection of the roots, the plant limits the endophytic colonization of *Trichoderma* through the activation of certain plant defense responses, including cell wall reinforcement and the accumulation of antimicrobial compounds and reactive oxygen species (Yedidia et al., 1999, 2000; Chacón et al., 2007; Contreras-Cornejo et al., 2011; Salas-Marina et al., 2011). After the successful limitation of fungus penetration to the first few layers of root cortical cells, the expression of some defense-related genes and the antimicrobial activity return to pre-infection levels (Yedidia et al., 1999, 2003; Masunaka et al., 2011). It is likely that *Trichoderma* is able to “ short-circuit” plant defense signaling, possibly through the secretion of still unknown fungal effectors, which suppress plant defense to remain accommodated by the plant as an avirulent symbiont. The interaction between the plant and *Trichoderma* should then be finely regulated, assuring benefits to both partners, with the plant receiving protection and more available nutrients and the fungus obtaining organic compounds and a niche for growth.

*Trichoderma* colonization triggers, therefore, a wide array of plant responses which may result in an enhanced defensive capacity of the plant (Bailey et al., 2006; Marra et al., 2006; Alfano et al., 2007; Morán-Diez et al., 2012). Often, the effects of *Trichoderma* on the plant defense system are not restricted to the root, but they also manifest in aboveground plant tissues (Martínez-Medina et al., 2010, 2011a; Salas-Marina et al., 2011; Mathys et al., 2012), rendering the plant more resistant to a broad-spectrum of plant pathogens. This systemic resistance is likely the result of the modulation of the plant defense network that may translate *Trichoderma-*induced early signaling events into a more efficient activation of defense responses. It is well known that the phytohormones jasmonic acid (JA), salicylic acid (SA), abscisic acid (ABA), and ET act as dominant primary signals in the regulation of local and systemic defense responses in plants (reviewed in Pieterse et al., 2009), and accordingly, they play a central role in the induced resistance phenomena. Generally, pathogen-induced systemic acquired resistance (SAR), is dependent on the SA-regulated signaling pathway (Durrant and Dong, 2004), while ISR by beneficial microorganisms usually relies on JA signaling (Pieterse et al., 1996; Van Loon et al., 1998; Pozo et al., 2008; Van Wees et al., 2008; Van der Ent et al., 2009). However, as more resistance-inducing agents are characterized, the implication of other signaling pathways in the induction of resistance becomes evident. Indeed is the cross-talk among different signaling pathways what provides the plant with a powerful capacity to finely regulate its immune response to specific invaders (Pieterse et al., 2009), and as induced resistance is usually an enhancement of basal defenses, the implication of multiple hormones in shaping ISR is likely. Induced resistance may result of the direct activation of defense mechanisms – including increased basal levels of defense-related hormones, or of the priming of the plant defensive capacity. In the latter, a more efficient activation of defense mechanisms occurs upon attack, and it may not be related to changes in hormone content but in the susceptibility of the tissues to these hormones (Conrath et al., 2006).

Expression studies on marker genes linked to the main defense signaling pathways suggested that *Trichoderma*-induced systemic resistance (TISR) might involve the direct activation of both SA- and JA-related pathways (Alfano et al., 2007; Salas-Marina et al., 2011; Mathys et al., 2012; Morán-Diez et al., 2012). Despite this possible direct activation of defenses, most examples points to a boosted activation of defenses upon attack by several pathogens (Segarra et al., 2009; Perazzolli et al., 2011; Brotman et al., 2012; Mathys et al., 2012). Nevertheless, the activation of a pathway does not proof its role in resistance. The requirement of a specific signaling pathway in TISR can only be addressed by phenotypic studies of disease development on mutant lines impaired in those pathways, however, only a limited number of studies in the model plant *Arabidopsis* have addressed this issue. The pioneer study by Korolev et al. (2008) using multiple *Arabidopsis* mutant lines showed that the induction of resistance by *Trichoderma harzianum* Rifai T39 against *Botrytis cinerea* requires JA, ET, and ABA signaling, while SA was not required. Using different *Trichoderma* strains and the same *Arabidopsis*–*B. cinerea* pathosystem other authors have confirmed the requirement of JA for TISR, while the need of an intact SA and ET signaling pathways is more controversial (Segarra et al., 2009; Mathys et al., 2012). In summary, in *Arabidopsis* JA has been consistently reported as essential for TISR against *B. cinerea* and other pathogens, but the requirement of SA and ET may depend on the *Trichoderma* strain (Korolev et al., 2008; Segarra et al., 2009; Mathys et al., 2012).

According to the reported data, it is likely that the induction of resistance against specific pathogens in different hosts may require different signaling pathways. Although induction of TISR in tomato has been demonstrated against bacterial and fungal pathogens (Alfano et al., 2007; Tucci et al., 2011), the signaling pathways involved are yet to be investigated. Here we aim to gain further insights in the role of the main defense signaling pathways that operate in TISR in tomato against the major fungal pathogen *B. cinerea* (Dean et al., 2012). First we try to uncouple the role of plant defense mechanisms from the possible contribution of nutritional aspects. Then we analyzed the signaling pathways required for efficient TISR establishment through the phenotypic analysis of disease on tomato signaling mutants. Finally, we explore the plant defense response triggered upon pathogen attack in induced plants by monitoring the expression of defense-related marker genes.

In summary, we present an integrative analysis of the main mechanisms implicated in the systemic resistance induced by *T. harzianum* T-78 in an agronomically important crop, tomato, against the gray mold causal agent *B. cinerea*. The hormonal related pathways implicated in TISR have been analyzed in order to provide insights into the signaling network regulating systemic resistance induced by *Trichoderma*in tomato.

## MATERIALS AND METHODS

### MICROBIAL STRAINS AND INOCULA PREPARATION

*Trichoderma harzianum* T-78 (CECT 20714, Spanish collection of type cultures) inoculum was prepared using a specific solid medium, obtained by mixing commercial oat, bentonite, and vermiculite according to Martínez-Medina et al. (2009). The necrotrophic fungus used in this study was *B. cinerea* CECT2100 (Spanish collection of type cultures) kindly provided by Dr. Flors (Universidad de Valencia). For spore production, *B. cinerea* was cultured on potato dextrose agar (PDA; Difco Laboratories, Detroit) supplemented with tomato leaves at 40 mg ml^-^^1^ at 24°C (Vicedo et al., 2009). *B. cinerea* spores were collected from 15-day-old cultures and incubated in Gambor’s B5 medium (Duchefa, Haarlem, The Netherlands) supplemented with 10 mM sucrose and 10 mM KH_2_PO_4_ for 2 h in the dark with no shaking, according to Vicedo et al. (2009).

### PLANT MATERIAL

Ten different tomato (*Solanum lycopersicum*) genotypes were used in our studies including the four wild-type cultivars Castlemart, Moneymaker, UC82B, and Betterboy and the following defense-related mutant lines: The JA-impaired mutant *def1* (Howe et al., 1996) in background Castlemart (provided by G. Howe, Michigan State University). The SA- and ABA-impaired lines *NahG* (Brading et al., 2000) and *sitiens* (Taylor et al., 1988) respectively, in background Moneymaker (provided by J. Jones, John Innes Centre and C. Hanhart, Wageningen University, respectively). The ET-impaired mutant *ACD* (Klee et al., 1991), in background UC82B (provided by H. Klee, University of Florida). The prosystemin antisense line *PS-* (Orozco-Cardenas et al., 1993) and the over-expressing line *PS+* (McGurl et al., 1994) both in background Betterboy (provided by C. Ryan and G. Pearce, Washington State University). Seeds were surface-sterilized in 4% sodium hypochlorite containing 0.02% (v/v) Tween-20, rinsed thoroughly with sterile water and germinated for 1 week in sterile vermiculite at 25°C in darkness.

### EXPERIMENTAL DESIGN AND GROWTH CONDITION

Individual seedlings were transferred to 0.25 l pots with a sterile sand:soil (4:1) mixture containing the *Trichoderma* inoculum. *T. harzianum* inoculum was mixed through the soil to a final density of 1 × 10^6^ conidia per g of soil before transplanting the tomato seedlings. The same amount of sand:soil mix but free from *T. harzianum* was added to control plants. For each treatment a total of six plants were used. Plants were randomly distributed and grown in a greenhouse at 24/16°C with a 16/8 h photoperiod and 70% humidity, and watered three times a week with Long Ashton nutrient solution (Hewitt, 1966). After 5 weeks, plants were harvested and the roots and shoot fresh weights were determined. The fourth and fifth leaves of each plant were detached for inoculation with the pathogen, and the rest of the shoots reserved for nutritional analyses. Root samples of each individual plant were thoroughly rinsed and collected for microbiological analyses. Substrate attached to the root system was considered as rhizospheric substrate and reserved for microbiological analyses.

### *Botrytis cinerea* BIOASSAY

The fourth and fifth leaves of each individual plant were detached from the plant with a blade and challenged with the pathogen by applying 5-μl droplets of a suspension of *B. cinerea* spores at 5 × 10^6^ ml^-^^1^, previously incubated in Gambor’s B5 medium supplemented with sucrose (0.1 mM) and phosphate (0.1 mM) for 4 h (Vicedo et al., 2009). One leaflet of each detached leaf from control and *T. harzianum*-inoculated plants were collected and immediately frozen in liquid nitrogen and stored at -80°C until use in molecular analyses as uninfected controls (time 0). Two 5-μl droplets were applied on each of the remaining leaflets, one on each side of the midrib. Detached *Botrytis*-inoculated leaves were placed on wet paper within plastic trays covered with transparent film to maintain high relative humidity conditions, and kept at 15–20°C with a photoperiod of 16 h light. Fungal hyphae grew concentrically from the inoculation site, resulting in visible necrosis at 48 h after inoculation. Disease symptoms were scored 72 and 96 h post inoculation (hpi) by determining the average lesion diameter in 12 leaves per genotype and treatment.

### PLANT NUTRIENT CONTENT ANALYSES

Nutrient content of shoots was measured at CEBAS-CSIC (Spain). Leaves were briefly rinsed with deionized water and oven-dried at 60°C for 72 h, and ground to a fine powder. The samples were digested by a microwave technique, using a Milestone Ethos I microwave digestion instrument, according to Martínez-Medina et al. (2011b). A standard aliquot (0.1 g) of dry, finely ground plant material was digested with concentrated nitric acid (HNO_3_; 8 mL) and hydrogen peroxide (H_2_O_2_; 2 mL). Subsequently, plant content of nutrition elements, including phosphorus and potassium, were simultaneous analyzed using ICP (Iris intrepid II XD2 Thermo). Nitrogen content was determined using a Flash 1112 series EA carbon/nitrogen analyzer. Six biological replicates from six independent plants were measured for each treatment.

### *Trichoderma* QUANTIFICATION IN THE RHIZOSPHERE

Serial dilutions of the sand:soil mixture samples in sterile, quarter-strength ringer solution were used for quantifying *T. harzianum* colony forming units (cfu), by a plate count technique using PDA amended with 50 mg L^-^^1^ rose bengal and 100 mg L^-^^1^ streptomycin sulfate, according to Martínez-Medina et al. (2011b). Plates were incubated at 28°C and cfu were counted after 5 days. Data were expressed per gram of dry soil.

### ANALYSIS OF GENE EXPRESSION BY RT-qPCR

Total RNA from tomato leaves was extracted using Tri-Reagent (Sigma-Aldrich) according to the manufacturer’s instructions. The RNA was treated with RQ1 DNase (Promega), purified through a silica column using the NucleoSpin RNA Clean-up kit (Macherey-Nagel), and stored at -80°C until use. Leaf tissue was collected from tomato leaves 96 h upon pathogen infection. The second leaflet of the leaves also was collected as uninfected control. The complementary DNA (cDNA) synthesis, the conditions of RT-qPCR (reverse transcription-quantitative polymerase chain reaction) experiments and the relative quantification of specific mRNA levels was performed according to López-Ráez et al. (2010) and using the gene-specific primers described in **Table 1**. Expression values were normalized using the housekeeping gene *SlEF*, which encodes for the tomato elongation factor-1α. The experiments were independently repeated and each reaction was performed in duplicate.

**Table 1:** Primer sequences used in the gene expression analysis. The genes monitored are used as markers for the pathways indicated. Jasmonate (JA), salicylic acid (SA), abscisic acid (ABA), and ethylene (ET)

ID	Target Gen	Related pathway	Primer (5′–3′)
AF083253	Multicystatin^1^ (*MC*)	JA inducible	GAGAATTTCAAGGAAGTTCAA
			GGCTTTATTTCACACAGAGATA
K03291	Proteinase inhibitor II^1^(*PI II*)	JA inducible	GAAAATCGTTAATTTATCCCAC
			ACATACAAACTTTCCATCTTTA
M84801	Prosystemin^2^(*PS*)	JA and ABA inducible	AATTTGTCTCCCGTTAGA
			AGCCAAAAGAAAGGAAGCAAT
M69247	Pathogenesis-related protein PR1a^3^ (*PR1*)	SA inducible	GTGGGATCGGATTGATATCCT
			CCTAAGCCACGATACCATGAA
M83314	Phenylalanine ammonia lyase^2^ (*PAL*)	SA biosynthesis	CGTTATGCTCTCCGAACATC
			GAAGTTGCCACCATGTAAGG
X51904	Desiccation protective protein^3^ (*Le4*)	ABA inducible	ACTCAAGGCATGGGTACTGG
			CCTTCTTTCTCCTCCCACCT
NM001247876	β-1,3-glucanase^2^ (*gluB*)	ET inducible	CCATCACAGGGTTCATTTAGG
			CCATCCACTCTCTGACACAACT
X14449	Elongation factor 1 α ^4^ (*SlEF*)	Housekeeping	GATTGGTGGTATTGGAACTGTC
			AGCTTCGTGGTGCATCTC
XM_001560987.1	β-tubulin^5^	Quantification of *B. cinerea*tubulin mRNA levels	CCGTCATGTCCGGTGTTACCAC
			CGACCGTTACGGAAATCGGAA

1 Uppalapati et al. (2005)

2 This work

3 López-Ráez et al. (2010)

4 Rotenberg et al. (2006)

5 Brouwer et al. (2003)

### STATISTICAL ANALYSES

The statistical analyses were performed using SPSS software, version 20 (SPSS Inc., Chicago, IL, USA). The data on lesion diameter in different tomato genotypes were subjected to two-way analysis of variance (ANOVA). The statistical significance of the results was determined by performing Tukey’s multiple-range test (*P* < 0.05). For data on plant nutritional content, pairwise comparisons were made for each genotype between *Trichoderma*-inoculated and control plants with Student’s *t*-test (*P* < 0.05). Regarding *T. harzianum* quantification in soil, the non-inoculated treatments were excluded from the analyses since *T. harzianum* was not detected in any of the non-inoculated treatments, and pairwise comparisons were made between each impaired mutant and its corresponding wild-type with Student’s *t*-test (*P* < 0.05). For gene expression analyses in the wild-type Moneymaker, pairwise comparisons were made for each gene between *Trichoderma*-inoculated and control plants with Student’s *t*-test (*P* < 0.05). Pairwise comparisons with Student’s *t*-test (*P* < 0.05) were also made for expression analysis between *Trichoderma*-inoculated and control plants for each gene in the genotypes *def1* and Moneymaker. All the experiments were repeated at least 2 times, with similar results.

## RESULTS

### *Trichoderma harzianum* INDUCES SYSTEMIC PROTECTION AGAINST *Botrytis cinerea* INFECTION

**FIGURE 1:**
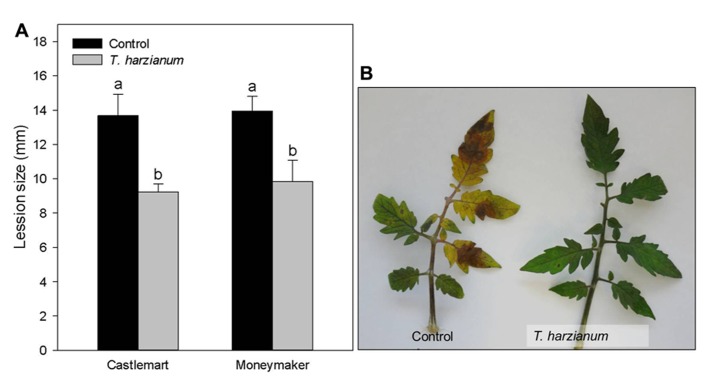
***Trichoderma harzianum* induces systemic protection against the pathogen *Botrytis cinerea* in tomato plants.(A)** Leaves of 5 weeks-old tomato plants (cv. Castlemart and Moneymaker) grown in soil containing or not *T. harzianum* were challenged with a conidial suspension of *B. cinerea*. Lesion diameter was determined 96 h after pathogen inoculation. The data show the lesion diameter (mm) ±SE (n = 12). Data not sharing a letter in common differ significantly (*P* < according to Tukey’s multiple-range test. **(B)** *B. cinerea* symptom development in *T. harzianum* inoculated and non-inoculated (control) plants (cv. Moneymaker).

Five-weeks old plants of two different tomato cultivars (Castlemart and Moneymaker) inoculated with *T. harzianum* were challenged with the foliar pathogen *B. cinerea*. The progress of the disease was recorded and data corresponding to 96 hpi are shown. *T. harzianum*-inoculated plants resulted in a statistically significant reduction of lesion diameter in both cultivars,compared with untreated control plants (**Figures 1A,B**).

### THE SYSTEMIC PROTECTION TRIGGERED BY *Trichoderma harzianum* IN TOMATO IS NOT RELATED TO IMPROVED NUTRITION OR GROWTH PROMOTION

In order to determine the effect of *T. harzianum* on plant development, shoot and root fresh weighs were evaluated and nitrogen, phosphorous, and potassium shoot content were measured on the tomato lines Castlemart and Moneymaker 5 weeks after inoculation with *T. harzianum*. There were no significant differences in growth associated to *T. harzianum* inoculation in any of the tomato lines (**Table 2**). Except for a moderate decrease in potassium levels in Castlemart, the nutrient analyses in shoots showed no differences in the main macronutrients nitrogen and phosphorous between *Trichoderma*-inoculated and control plants, suggesting that *Trichoderma* effects on disease development cannot be regarded as a consequence of improved plant growth or nutrition improvement.

**Table 2:** Effect of *Trichoderma harzianum* on tomato plant development. Shoot and root fresh weight (in grams) and shoot nitrogen, phosphorous, and potassium content (g/100 g fresh weigh) of 5-weeks old tomato lines Castlemart and Moneymaker inoculated with *T. harzianum*.

Tomato Cv.	Treatment	Shoot fresh weight (g)	Root fresh weight (g)	Shoot nitrogen (g/100 g)	Shoot phosphorous (g/100 g)	Shoot potassium
Castlemart	Control	9.90± 0.46	1.63 ± 0.16	2.69 ± 0.20	0.243 ± 0.054	2.54 ± 0.13	
	*T. harzianum*	8.20 ± 0.30	1.67 ± 0.35	2.43 ± 0.21	0.174 ± 0.012	2.05 ± 0.09*
Moneymaker	Control	10.15 ± 0.57	1.77 ± 0.15	1.90 ± 0.14	0.164 ± 0.045	2.30 ± 0.09
	*T. harzianum*	10.05 ± 0.69	1.32 ± 0.19	2.02 ± 0.34	0.124 ± 0.008	2.66 ± 0.24

The data are the means of six replicates ± SE. For each tomato genotype asterisks indicate statistically significant differences between *T. harzianum* inoculated and non-inoculated plants (Student’ s *t*-test, *P* < 0.05).

### *Trichoderma harzianum*-INDUCED SYSTEMIC RESISTANCE IS DEPENDENT ON THE PHYTOHORMONES JA, SA, AND ABA

**FIGURE 2:**
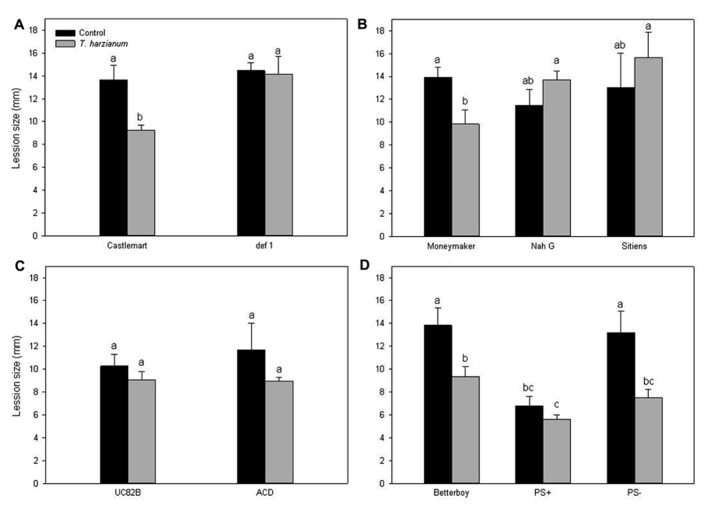
***Trichoderma*-induced systemic resistance requires JA, SA, and ABA. Lesion diameter was measured 96 h after challenge with the pathogen in (A)** the wild-type tomato plants cv. Castlemart and the JA-impaired mutant *def1*; **(B)** in the wild-type cv. Moneymaker and the SA- and ABA-impaired mutants *NahG* and *sitiens*, respectively; **(C)** in the wild-type cv. UC82B and in the ET-impaired mutant *ACD*; and **(D**) in the wild-type cv. Betterboy and in the over-expressing mutant line *PS+* and the prosystemin silenced line *PS-*. The data show the lesion diameter (mm) ± SE (*n* = 12). Data not sharing a letter in common differ significantly (*P* < 0.05) according to Tukey’s multiple-range test.

In order to analyze the involvement of different defense-related pathways in *Trichoderma*-mediated ISR, we investigated the effect of *T. harzianum* on *B. cinerea* infection in different tomato mutant lines and their corresponding backgrounds. Mutants affected in the biosynthesis of specific defense-related hormones were selected, including the JA-deficient *defenseless1* (*def1*), the SA-deficient *NahG*, the ABA-deficient *sitiens* and the ET-underproducing ACC deaminase *ACD*. Additionally, we also analyzed the disease development in the tomato lines over-expressing the *prosystemin* gene in the sense (*PS+*) and antisense (*PS-*) orientation. Prosystemin is the precursor of the peptide defense hormone systemin, a positive regulator of JA signaling. The evaluation of the lesions upon *Botrytis* inoculation revealed that disease development was significantly affected by the plant genotype (*P* < 0.001; *F* = 7.43), the fungal treatment (*P* < 0.001; *F* = 10.98) and their interaction (*P* < 0.01; *F* = 2.82), as confirmed by two-way ANOVA analysis. As shown in **Figure 2A** the suppressive effect on *B. cinerea* disease observed in the wild-type Castlemart plants elicited with *T. harzianum* was absent in the JA-deficient *def1* mutant, indicating that JA-regulated pathway is required for TISR against *B. cinerea*. Similarly, the mutant lines impaired in SA (*NahG*) and ABA (*sitiens*) accumulation did not display the TISR against *B. cinerea* observed in their corresponding background Moneymaker (**Figure 2B**). In the transgenic *NahG* line SA-accumulation is blocked through the transformation of SA to catechol. Interestingly, we observed a lower susceptibility of *NahG* control plants toward *B. cinerea* infection compared to its parental wild-type Moneymaker (*P* < 0.1), which support the idea that SA affect negatively basal resistance against this necrotroph in tomato (**Figure 2B**). In contrast to Castlemart and Moneymaker, the wild-type UC82B plants were unable to develop *T. harzianum* ISR (**Figure 2C**). In the ET-underproducing ACC deaminase mutants (*ACD*), *T. harzianum* slightly, but not significantly reduced the pathogen lesion (above 20%). Concerning systemin, plants of the over-expressing mutant line *PS+*, elicited and non-elicited with *T. harzianum* were more resistant to the necrotroph than any other cultivar tested (*P* < 0.05), confirming the involvement of this molecule in tomato basal resistance against *B. cinerea*. Although *T. harzianum*-induced resistance in the wild-type Betterboy, *Trichoderma* colonization could not reduce further *B. cinerea* disease development in *PS+*. Remarkably, *T. harzianum* was also able to induce ISR in the tomato line silenced in prosystemin expression *PS-* (**Figure 2D**).

### *Trichoderma harzianum* EFFECTIVELY COLONIZES THE RHIZOSPHERE AND ROOTS OF WILD-TYPE AND MUTANT TOMATO LINES

The biocontrol effect of *Trichoderma* is associated to its efficient colonization of the rhizosphere. To analyze if the unability of the mutants to mount TISR is related to a deficient *Trichoderma* colonization, we tested the ability of *T. harzianum* to colonize the rhizosphere of the different tomato mutant lines and their correspondent backgrounds. The number of *Trichoderma* colony-forming units (cfu) in the rhizosphere, determined after 5 weeks, was similar to initial inoculation values in all the tested lines. We did not find significant (*P* < 0.05) differences in cfu numbers in the rhizosphere of the different tomato mutant lines compared to their corresponding genetic backgrounds. Moreover, endophytic colonization was also confirmed for all of the lines. Incubation of surface-sterilized roots under appropriate conditions revealed that *Trichoderma* could outgrow from inside the roots regardless of the plant genotype. The results indicated that the impairment on the production of the hormones JA, SA, ABA, ET, or systemin does not affect *T. harzianum* capacity for rhizosphere and root colonization.

### *Trichoderma harzianum* PRIMES JASMONATE-DEPENDENT DEFENSES

**FIGURE 3:**
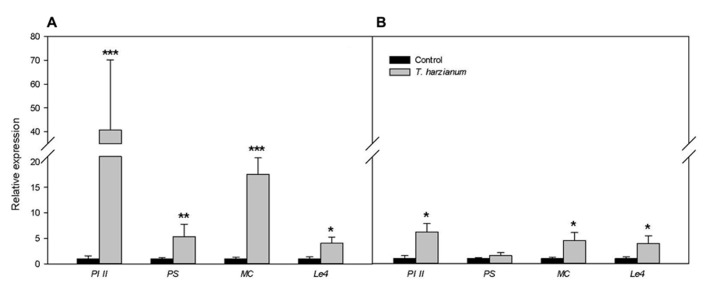
***Trichoderma* primes JA-regulated responses.** The expression of different defense-related marker genes was analyzed in *T. harzianum* inoculated and non-inoculated (control) plants (cv. Moneymaker) 96 h upon pathogen infection (A) and before infection **(B)**. Expression levels of the JA-related marker genes *PI II*, *MC*, and *PS*; and the ABA-related marker gene *Le4* is shown. The results were normalized to the *SlEF* gene expression levels. The expression levels are reported as the fold increase relative to that of the control plants not treated with *T. harzianum* ± SE (*n* = 5). Asterisks indicate statistically significant differences between *Trichoderma* induced and non-induced plants (Student’s *t*-test, *P < 0.05; **P < 0.01; ***P < 0.001).

Induced systemic resistance by beneficial microbes is commonly not associated with major changes in defense-related gene expression. Instead, a relatively mild systemic immune reaction is triggered that is frequently associated with priming for enhanced defense. In order to establish whether the enhanced resistance induced by *T. harzianum* in tomato was associated with priming of plant defense, we compared the plant response to *B. cinerea* in *Trichoderma* elicited and not elicited plants. We analyzed by RT-qPCR the expression of known marker genes for the main plant defense-related pathways in *B. cinerea* challenged plants. No significant differences were found for the marker genes of SA- (*PR1a* and *PAL*) or ET- (*gluB*) modulated pathways between *Trichoderma* induced and not induced plants (data not shown). In contrast, an enhanced expression of the JA responsive genes *PI II*, *MC*, and *PS*, coding for proteinase inhibitor II, multicystatin, and prosystemin, respectively, was found in *T. harzianum*-elicited compared to non-elicited plants (**Figure 3A**). Interestingly, *T. harzianum*-inoculated plants showed no or slight induction of those genes in the absence of the pathogen (**Figure 3B**), thus pointing at priming of the JA-dependent defense responses as the mechanism underlying the induction of resistance against *B. cinerea*. *T. harzianum*-colonized plants also displayed higher levels of expression of the ABA responsive marker gene *Le4* (coding for a desiccation protective protein) after pathogen challenge, but a similar increase was observed in *T. harzianum* induced plants in the absence of the pathogen (**Figures3A,B**).

**FIGURE 4:**
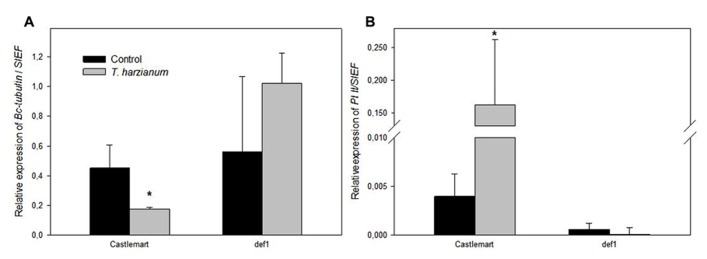
***Trichoderma harzianum* priming of defenses requires JA signaling.** The *Botrytis cinerea* constitutive gene *Bc*-*Tubulin* **(A)**, and the JA-related marker gene *PI II* **(B)** were analyzed in leaves of the wild-type tomato plants cv. Castlemart and the JA-impaired mutant *def1* upon 96 h of *B. cinerea* infection. Results were normalized to the *SlEF* gene expression in the same samples. Data show the relative expression level (±SE). For each tomato genotype asterisks indicate statistically significant differences between *Trichoderma* induced and non-induced plants (Student’s *t*-test, *P* < 0.05, *n* = 5).

We further confirmed the priming of the JA-dependent defense responses against *B. cinerea* with the analysis of pathogen proliferation and the induction of JA responses in leaves of the wild-type Castlemart and the JA-deficient *def1*. Expression levels of a *B. cinerea* constitutive gene in leaves confirmed that the differences observed in symptom development (**Figure 2**) were due to differences in pathogen proliferation in the tissues, and confirmed that *def1* plants were unable to develop *Trichoderma*-induced resistance in contrast to the wild-type Castlemart (**Figure 4A**). The inability of *def1* plants to develop TISR correlated with a lack of priming of *PI II* expression (**Figure 4B**) further supporting the essential role of primed JA responses in the enhanced systemic resistance triggered by *Trichoderma*.

## DISCUSSION

Selected *Trichoderma* species colonize plant roots and establish symbiotic relationships with the plant. As consequence, plant resistance against pathogens is frequently enhanced, even in aboveground tissues (Segarra et al., 2009; Fontenelle et al., 2011; Perazzolli et al., 2011; Brotman et al., 2012; Yoshioka et al., 2012). In this study we analyzed the effectiveness of *T. harzianum* T-78 root colonization in the enhancement of tomato resistance against the foliar necrotrophic pathogen *B. cinerea*. *T. harzianum* T-78 is an effective biocontrol agent in the soil (Martínez-Medina et al., 2011b), with high mycoparasitic capacity (López-Mondéjar et al., 2011), but its ability to induce plant resistance was not previously tested.

We found that treatment of tomato roots with *T. harzianum* T-78 clearly reduced disease development upon inoculation with the necrotrophic pathogen *B. cinerea* in three out of four cultivars tested (Castlemart, Moneymaker, and Betterboy). We examined the presence of *Trichoderma* isolate T-78 in the shoots and we could not detect its presence in any of the tomato cultivars (data not shown). Therefore, *Trichoderma* and the pathogen remain physically separated, and accordingly, it can be concluded that *T. harzianum* T-78 activates a plant-mediated systemic response that is effective in restricting *B. cinerea* development. The dependence on the plant genotype of TISR against *Botrytis*, also shown for other tomato cultivars (Tucci et al., 2011), further confirms that the protection depends on plant-mediated mechanisms. Other studies have shown the ability of different *Trichoderma* strains to induce a plant-mediated effect against this necrotroph, mostly in *Arabidopsis* (Korolev et al., 2008; Contreras-Cornejo et al., 2011; Mathys et al., 2012) but also in other crop plants (De Meyer et al., 1998; Tucci et al., 2011).

*Trichoderma* colonization is reported to improve plant nutrition and growth in several plant species (Martínez-Medina et al., 2011b; Salas-Marina et al., 2011; Tucci et al., 2011). Since improved plant nutritional is considered one of the mechanisms responsible for bioprotection against pathogens by beneficial microorganisms (Whipps, 2001) we tried to analyze the contribution of this effect to the enhanced resistance observed. In our experimental conditions there was no increase in plant growth or nutrient content associated to *Trichoderma* colonization, probably because plants were grown under optimal conditions (Martínez-Medina et al., 2011b). Thus, our experimental system allows uncoupling nutritional from defense effects, and it can be concluded therefore that the protective effect observed in *Trichoderma* T-78-inoculated plants was related to mechanisms other than an improved nutrition, most likely related to plant defenses.

As for ISR by selected non-pathogenic rhizobacteria (Van Wees et al., 1999; Verhagen et al., 2004; Pozo et al., 2008), some studies have shown that the systemic resistance triggered by *Trichoderma* requires responsiveness to JA and ET (Shoresh et al., 2005; Segarra et al., 2009; Perazzolli et al., 2011; Tucci et al., 2011). However, phenotypic analysis of disease on *Arabidopsis* signaling mutants revealed that other small-molecule hormones such as SA or ABA could also play pivotal roles in the regulation of this network (Korolev et al., 2008; Mathys et al., 2012; Yoshioka et al., 2012). To determine the main signaling pathways involved in the induced systemic resistance elicited by *T. harzianum* T-78 in tomato against *B. cinerea*, we assessed the ability of different hormone-impaired tomato mutants for TISR development. The phenotypic analysis of disease development in the JA (*def1*)- and SA (*NahG*)-impaired mutants demonstrated that *T. harzianum*-induced systemic resistance against *B. cinerea* requires not only the JA but also the SA signaling pathways, as these mutant lines developed similar level of disease than non-induced control plants. Similarly, a recent study showed a role of the SA-pathway in *T. hamatum* T-382-induced ISR against *B. cinerea* in *Arabidopsis*, as TISR was blocked in the SA-impaired mutants *NahG* and *sid2* (Mathys et al., 2012). In contrast, *Trichoderma asperellum*-induced resistance in *Arabidopsis* against the hemibiotrophic leaf pathogen *Pseudomonas syringae* seems independent of SA, as TISR was fully expressed in the SA-impaired mutant *sid2* (Segarra et al., 2009). Thus experimental evidences support that induced resistance is a flexible process that may involve different signaling pathways depending on the mode of action of the pathogen, as it has been shown for some resistance-inducing chemicals (Flors et al., 2008). Our results demonstrate that in tomato, both SA and JA signaling pathways are required for TISR development against *B. cinerea*. Necrotrophic pathogens are usually controlled by JA-defense responses (Glazebrook, 2005), and JA signaling has been shown as key for basal resistance to *Botrytis* in tomato (AbuQamar et al., 2008; El-Oirdi et al., 2011). It is therefore not surprising the requirement of intact JA-related hormonal signaling pathway for boosted plant defenses against *Botrytis* by *Trichoderma*. The role of the SA signaling in plant resistance against *B. cinerea* is, however, more complex (Ferrari et al., 2003). Recently it has been shown that SA plays a regulatory role in the balance between disease and resistance as *Botrytis* induces SA signaling to promote disease in tomato through its negative interaction with the JA-dependent pathway (El-Oirdi et al., 2011).

In relation to ET, since the wild-type plants UC82B were unable to develop *T. harzianum*-induced ISR, we were unable to determine if ET signaling is required for TISR against *B. cinerea*. In contrast to earlier findings in rhizobacteria-mediated ISR (Pieterse et al., 1998; Knoester et al., 1999), Mathys et al. (2012) observed a limited role of the ET pathway in *T. hamatum* T382-induced resistance. Our results, although inconclusive, are in line with this finding as a reduced disease development on ET-mutants (*ACD*) was observed. It is noticeable that non-induced wild-type UC82B plants showed the lowest susceptibility to *B. cinerea* among all cultivars tested, and likely *T. harzianum* was unable to further boost plant resistance.

Additionally, analysis of the disease development in the ABA-deficient mutant *sitiens* showed that disruption of the ABA signaling results in the loss of ability to develop TISR against *B. cinerea*. Although ABA is commonly associated with plant development and abiotic stress, its role in plant immunity is now clear, as this hormone has been shown to be connected to the SA–JA–ET network (Anderson et al., 2004; Mauch-Mani and Mauch, 2005). The role of ABA in tomato resistance against pathogens is controversial, and indications for both a role in susceptibility and resistance have been given (Flors et al., 2008; Sánchez-Vallet et al., 2012). In tomato, a negative regulatory role of ABA in resistance to *B. cinerea* has been proposed, as the *sitiens* mutant showed reduced susceptibility than wild-type plants (Audenaert et al., 2002; Asselbergh et al., 2007, 2008). In our system *sitiens* plants did not show enhanced basal resistance compared to wild-type plants, indicating that ABA is not a major player in basal resistance, but it is important for *Trichoderma*-induced resistance. In line with these observations, Vicedo et al. (2009) found that ABA-deficient mutants were not affected in basal resistance against *B. cinerea*, but they were impaired in hexanoic acid-mediated protection against this pathogen, also based in primed JA responses.

Finally, systemin has been also shown to play a role in resistance against *B. cinerea* in tomato (Díaz et al., 2002; El-Oirdi et al., 2011). The disease examination in the over-expressing *PS+* mutant line confirmed a role of the polypeptide in the basal resistance against *B. cinerea*, as over-expressing *PS+* mutants were highly resistant to the necrotroph. Probably because of this high resistance, *Trichoderma* was unable to boost further resistance in this line. Remarkably, the analysis of the line silenced in prosystemin expression *PS-* showed that TISR was fully expressed in the *PS*- mutants suggesting that *T. harzianum*-mediated systemic resistance against *B. cinerea* does not rely on systemin signaling.

*Trichoderma* effects on plant defenses have been related to the fungal ability for intercellular root colonization (Yedidia et al., 2000; Chacón et al., 2007; Djonovic et al., 2007; Velázquez-Robledo et al., 2011). Successful rhizosphere and root endophytic colonization by *T. harzianum* T-78 was confirmed for all genotypes. Accordingly, the defect in TISR observed in some of the mutants is not related to defects in colonization but to the requirement of the hormone in the regulation of the plant response to the pathogen. The above findings demonstrate that *T. harzianum*-mediated resistance against *B. cinerea* requires the JA-, SA-, and ABA-regulated pathways. Cross-talk between hormonal-related signaling pathways acts as a cost-efficient regulatory mechanism for inducible defense responses (reviewed in Pieterse et al., 2009), and our results suggest that cross-talk between JA, SA, and ABA signaling pathways is essential for the induction of resistance mechanisms by *Trichoderma* T-78 in tomato. Nevertheless, it remains to be determined if additional hormones such as auxin, gibberellin, cytokinin, and brassinosteroids may also contribute to the regulatory network behind *Trichoderma*-induced resistance to *B. cinerea*.

Once key elements in the regulation of the response during TISR were identified, we aim to identify the actual defense responses underlying the resistance in *Trichoderma*-inoculated plants. For that we compared the plant defense response to *Botrytis* infection in *Trichoderma* elicited and not elicited plants through the expression analysis of known defense genes, markers for the main defense-related pathways. *T. harzianum* colonization of the roots resulted in priming of the aboveground plant tissues for enhanced JA-responsive gene expression, as a boosted expression of the JA-regulated marker genes *PI II*, *PS*, and *MC* coding for the proteinase inhibitor II (Farmer and Ryan, 1992), prosystemin, the precursor of the hormone systemin (Farmer and Ryan, 1992) and multicystatin (Girard et al., 2007) was observed in *Trichoderma*-induced plants, upon *B. cinerea* infection. It has been recently reported that the proteinase inhibitor II encoded for *PI II* plays a major role for tomato resistance against *B. cinerea* (El-Oirdi et al., 2011). The induction of those genes in plant shoots by *Trichoderma* was relatively weak before *Botrytis* infection, thus pointing to priming of the JA-dependent defense responses as the mechanism underlying the induction of resistance against *B. cinerea*. Activation of a JA-related priming state in plants by *Trichoderma* has been observed previously in *Arabidopsis*, tomato, and grapevine plants (Segarra et al., 2009; Tucci et al., 2011; Brotman et al., 2012; Perazzolli et al., 2012) with no obvious costs for the plant. Indeed, priming by beneficial microorganisms offers broad-spectrum protection whenever required (Van der Ent et al., 2009), without significant energy costs to plant metabolism and growth (Walters and Heil, 2007; Van Wees et al., 2008). *Trichoderma* elicitation, however, did not boost SA- or ET-related defense responses against *B. cinerea*, as no variation in the SA-marker genes *PR1a* and *PAL* nor in the ET regulated *gluB* were found in our study, in contrast to earlier studies (Yedidia et al., 2003; Shoresh et al., 2005). Nevertheless, as resistance to *B. cinerea* is JA-dependent (AbuQamar et al., 2008) and SA signaling is the target of the pathogen to interfere with JA-signaling and promote disease (El-Oirdi et al., 2011), priming of SA responses in this interaction would be detrimental for the plant. Notably, *Trichoderma* inoculation induced the expression of the ABA-marker gene *Le4* before *B. cinerea* infection, suggesting a moderate direct activation of ABA-signaling that could participate in the defense against the pathogen.

The identification of primed JA responses as the control mechanism underlying TISR in tomato- *B. cinerea* pathosystem was further corroborated in the JA-impaired mutant *def1* through the quantification of pathogen biomass and the induction of plant defenses. The failure of *def1* plants to develop TISR correlated with a lack of priming for *PI II* expression. These results confirm the essential role of the boosted expression of JA responses in the enhancement of resistance by *Trichoderma*against *B. cinerea*.

**FIGURE 5:**
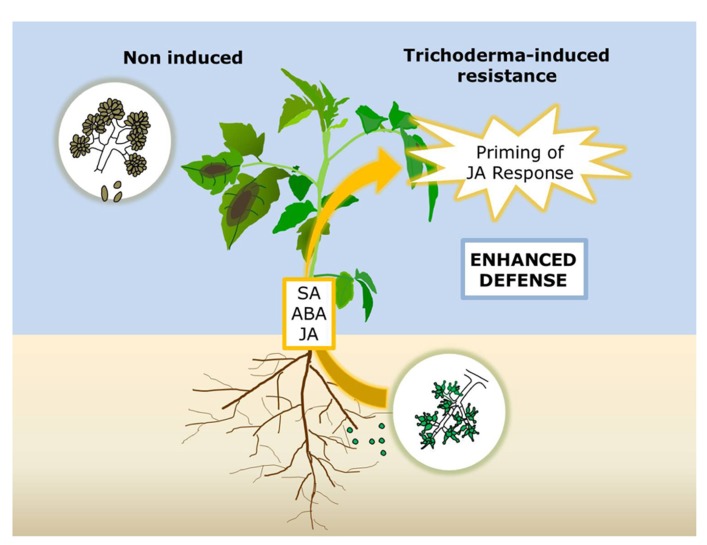
**Model for *Trichoderma-*induced resistance (TISR) against *Botrytis cinerea* in tomato.** Root colonization with *Trichoderma* primes leaf tissues for enhanced activation of JA-regulated defense responses leading to a higher resistance to the necrotroph. Intact JA, SA, and ABA signaling pathways are required for TISR development.

In summary, this study provides evidence that *T. harzianum* induces systemic resistance against *B. cinerea* in tomato through a boosted JA-dependent defense response, which reduce pathogen proliferation and disease development in plant leaves. The regulation of the response requires not only JA but also at least SA and ABA signaling (**Figure 5**). All in all, our results support the consistent central role of JA in the induction of resistance by different *Trichoderma* strains, and illustrate the requirement of other signaling pathways probably shaping the final response adapted to the challenging pathogen.

## Conflict of Interest Statement

The authors declare that the research was conducted in the absence of any commercial or financial relationships that could be construed as a potential conflict of interest.
